# Developing public health policy for addressing PCOS is hampered by the paucity of high-quality epidemiologic studies

**DOI:** 10.1210/jendso/bvaf200

**Published:** 2025-12-08

**Authors:** Ricardo Azziz, May Rudd

**Affiliations:** Department of Obstetrics & Gynecology, Heersink School of Medicine, University of Alabama at Birmingham (UAB), Birmingham, AL 35249, USA; Department of Medicine, Heersink School of Medicine, UAB, Birmingham, AL 35294, USA; Department of Healthcare Organization & Policy, School of Public Health, UAB, Birmingham, AL 35294, USA; Department of Health Policy, Management, and Behavior, School of Public Health, University at Albany, SUNY, Rensselaer, NY 12222, USA; Foundation for Research and Education Excellence, Vestavia Hills, AL 35243, USA; Department of Obstetrics & Gynecology, Heersink School of Medicine, University of Alabama at Birmingham (UAB), Birmingham, AL 35249, USA; Foundation for Research and Education Excellence, Vestavia Hills, AL 35243, USA

**Keywords:** PCOS, polycystic ovary syndrome, epidemiology, public health, policy, prevalence

Ramphul and colleagues used spatial analysis, leveraging the Texas Public Use Data File (PUDF) from 2018 to 2024, to identify polycystic ovary syndrome (PCOS)-related visits (ICD-10: E28.2) with the aim of identifying geographic areas of PCOS underdiagnosis (ie, cold spots) in the state [1]. The investigators noted that cold spots were concentrated in rural and peri-urban areas, suggesting potential underdiagnosis in these communities. Although the exact reason for the underdiagnosis could not be ascertained, the researchers noted that the data highlighted the need for targeted public health interventions, increased diagnostic outreach in rural settings, and greater provider training. The work of Ramphul and colleagues is highly commendable in striving to uncover gaps in PCOS diagnosis and care. By identifying geographic disparities, this study stresses the importance of considering the environment in the access to care for women with PCOS. Furthermore, the study highlights the complexity of studying populations with such mixed and complex ancestry as are the populations of Texas. This investigator cannot agree more with these assertions.

Targeted public health intervention begins with high-quality epidemiologic data. In 1998, we published the first report on the prevalence of PCOS in a medically unbiased population [2]. Before that study and those that followed, most if not all clinicians believed PCOS to be a somewhat infrequent and exotic disorder. Subsequent studies have reported population prevalence rates generally ranging from 8% to 13%. However, there is a notable paucity of high-quality epidemiologic data [3], including that arising from nonurban areas. In fact, even the prevalence estimates of PCOS in the United States are based on just 4 high-quality studies, primarily studying subjects in urban areas [3].

Careful epidemiologic studies in different regions and environments are critical because only they can reveal the impact of external factors on the development and phenotype of PCOS, including environmental, dietary, socio-economic, and ethnic factors. To address this deficiency, we developed a series of investigations across different geographic regions and ethnicities around the world, including Siberia, Nigeria, Ghana, Colombia, Trinidad-Tobago, and urban and rural Alabama: the PCOS Epidemiology and Phenotype (PEP) studies. PEP studies use the same investigational methods and techniques to increase data harmonization and comparability. In addition, we established the PCOS Phenotypes in Unselected Populations (P-PUP) study, to compare the PCOS phenotype across different countries, albeit understanding that there is some variation in assessment methods used [4]. Greater emphasis on obtaining high-quality epidemiologic data in varying populations is critically needed.

The frequent approach of using medically related records, be it private or governmental payers or provider electronic health records, to estimate the prevalence and phenotype of PCOS in the general population is problematic. These data primarily reflect access, patient urgency, and clinician acumen. In a recent report we observed that the prevalence of PCOS in health records was 5 to 15 times lower than the actual prevalence in the general population [5]. In another study we observed that the proportion of non-Hispanic White women with PCOS seen in the clinical setting was markedly higher than the proportion of women with this ethnicity among those diagnosed in a medically unbiased population, potentially reflecting the more limited access to healthcare that people of color experience [6]. Likewise, Ramphul et al presented data suggesting significant underdiagnosis of the disorder in nonurban areas [1]. However, we should be clear that the underdiagnosis, and even the misdiagnosis, of PCOS is relatively widespread negatively impacting PCOS patient satisfaction with their clinical care.

Public health policies improve population health outcomes and reduce health disparities by influencing prevention, treatment, and community health approaches for diseases and disorders. Public health policies do so by leveraging data which fosters the establishment of programs, education, regulations, and laws. The development of good public health policies for early detection and intervention require high-quality epidemiologic data. Some clinicians and investigators have the impression that the diagnosis of PCOS is complex. Though there are emerging genotype and machine-learning based studies attempting to identify subtypes in the disorder [7], we should note that recognizing PCOS by its clinical presentation is neither difficult nor complicated, including in population-based studies. Even self-reported excess body hair growth and menstrual dysfunction have a very high positive and negative predictive value for the disorder [8]. What is needed now is greater investment in epidemiologic research to obtain the critically needed public health data.

## Abbreviation

PCOS: polycystic ovarian syndrome
